# The Influence of Aligned MHD on Engine Oil-Based Casson Nanofluid with Carbon Nanotubes (Single and Multi-Wall) Passing through a Shrinking Sheet with Thermal Radiation and Wall Mass Exchange

**DOI:** 10.3390/mi13091501

**Published:** 2022-09-09

**Authors:** Irfan Rashid, Tamour Zubair, Muhammad Imran Asjad, Elsayed M. Tag-Eldin

**Affiliations:** 1 Department of Engineering and Computer Science, National University of Modern Languages, Islamabad 44000, Pakistan; 2 School of Mathematical Sciences, Peking University, Beijing 100871, China; 3 Department of Mathematics, University of Management and Technology, Lahore 54770, Pakistan; 4 Faculty of Engineering and Technology, Future University in Egypt, New Cairo 11835, Egypt

**Keywords:** heat transfer, nanofluid, wall mass transport, thermal radiations, aligned magnetic field, CNTs (single and multi-wall)

## Abstract

The optimization of heating or cooling during an industrial system may result in power savings, reduced processing time, enhanced thermal efficiency, and increased equipment operating lifespan. The advancement of high-efficiency thermal systems for heat and mass transport improvement has become increasingly popular in recent years. The analysis of aligned magnetohydrodynamics (MHD) on engine oil-based Casson nanofluid with carbon nanotubes (single and multi-wall) passing a shrinking sheet following the thermal radiation and wall mass transport phenomena is carried out in this aspect. The dynamic model is utilized to reduce difficult ordinary differential equations into nondimensional forms, which are then analytically assessed. To study the repercussions of a physical parameter on the velocity field, skin friction at the wall, the stream pattern, the temperature distribution, isotherm, and the local Nusselt, numeric data and visualizations are generated. When the value of ϕ increases, the velocity field decelerates, and the velocity pattern of multi-walled CNTs drops considerably when compared to single-walled CNTs. The local Nusselt number is a decreasing function of *N* and ϕ and the opposite trend is shown for Pr. The local Nusselt number is a decreasing function of *N* and ϕ and the opposite trend is shown for Pr. The single-walled CNTs have a higher degradation rate as compared to multi-walled CNTs. It is found that higher temperature distribution occurs in the case of multi-walled CNT-based fluid as compared to single-walled CNT-based fluid.

## 1. Introduction

Carbon nanotubes (CNTs) have sparked a lot of interest due to their unusual structural and impressive mechanical, heat transport, and electrical capabilities. They have been utilized as fluid additives to boost thermal properties, which is among the most significant concerns in industry [1]. These are some of the best nanoadditives for forming nanomaterials, such as nanofluids for heat dissipation, and nanofluids used in several consumer and industrial items and (nanolubricants) diverse metallurgical settings can improve from the use of nanolubricants since they increase viscosity, enhance heat transport, and provide lubricant the necessary functionalities [2]. From this perspective, we utilized carbon nanotubes (single and multi-wall) to enhance the thermal attributes of the Casson fluid, which has many fascinating applications, for instance, agents that reduce drag and cooling, blood circulation modeling, food processing, and the paper industry [3]. Choi proposed the concept of nanofluids, which were empirically supported [4]. Recently, using ramping surface conditions including heating effect, the circulation of an EO-based Casson nanofluid comprising Molybdenum disulfide (MoS2) nanoparticles was investigated by Siddique et al. [5]. Mahato et al. [6] investigated the entropy inception of Casson nanofluid on a porous stretchable wall accompanying MHD. Consequences of inclined MHD due to the vertical exponential surface were examined by Ishtiaq and Nadeem [7]. An MHD energy-disorder measure of a nanofluid above a stretching surface implanted in porous media was examined by Qing et al. [8]. Souayeh and Gnaneswara analyzed a [9] comparison of chaotic radiative energy transport using an MHD Casson nanofluid passing through a thick needle. The influence of convective energy exchange, as well as MHD on Casson nanofluid flowing across a shrinking wall, was examined by Haq et al. [10]. Mustafa and Khan [11] investigated the formulation for Casson nanofluid fluid flows above a non-linearly expanding wall with magnetic field effects. Sodium Alginate Casson nanofluid naturally convecting across a rigid body accompanying MHD was analyzed by Alwawi et al. [12]. Nadeem et al. [13] introduced an analytical problem with optimal solutions for Casson-nano fluid oblique movement using convection boundary constraints. Arif et al. [14] investigated the engine oil-based Casson nanofluid through a ramped sheet. The entropy generation calculated analytically for a Casson nanofluid past a stretchable surface was examined by Abolbashari et at. [15]. Yahya et al. [16] observed an engine oil-based hybrid nanofluid flow due to a stretching wall.

Magnetohydrodynamic flowing fluid on a shrinking surface has recently gained importance due to its numerous uses within engineering and industry. MHD has engrossing applications such as boilers, metal processing, cooling systems, heating insulation, devices that store energy, biological transmission, and micro-MHD pumps [17]. In that context, it is crucial to examine the flow and energy transport of the MHD Casson nanofluid. Aly and Pop [18] examined the flow and heat transport of MHD with a convection boundary state across a permeable stretching/shrinking surface inside a hybridized nanofluid. A mixed nanofluid on a stretched surface performing MHD flow, dual results, and stability is illustrated by Lund et al. [19]. Above a permeable stretching/shrinking surface, the flow of MHD hybridized nanofluid including heat radiation impact is scrutinized by Yashkun et al. [20]. Mahabaleshwar [21] studied the surface mass transport parameter influence on MHD nanofluid due to stretching/shrinking surface. Examination of magnetohydrodynamic nanofluid circulation analytically inside a semi-porous channel was carried out by Sheikholeslami et al. [22].

According to the aforementioned interesting applications, this study’s main goal is to analyze the different effects of physical parameters on Casson nanofluid and enhance the thermal properties of conventional base fluid by incorporating small amounts of carbon nanotube (SWCNTs/MWCNTS) nanoparticles. We investigated the CNT engine oil-based nanofluid over a shrinking wall, accompanying the power law index and angled MHD effect. The analytical results are obtained. Additionally, we seek to comprehend the impact of different physical factors on fluid temperature, Nusselt number, velocity, and skin friction, so numerical tables, and figures are displayed. Moreover, the flow and heat transport patterns are depicted through stream lines and isotherm figures.

## 2. Novelty and Applications

The basic objective of this research is to improve the thermal properties of customary base fluid by incorporating limited portions of nanoparticles such as carbon nanotubes (SWCNTs/MWCNTS). Many researchers have studied the heat and mass transport of Newtonian nanofluids in the last few years. From the literature survey, no work has been carried out on heat and mass transport of non-Newtonian CNT engine oil-based Casson nanofluid with power law index, aligned MHD, wall mass exchange parameter, and thermal radiation past a shrinking wall through Kummer’s functions. Because of its novel applications in motor oils, blood, suspensions, lubricants, mining industries, and medicine, many researchers are studying the heat and mass transport of nanofluids. Every non-Newtonian fluid behaves differently and much recognition is required to examine the rheological properties of every dynamical fluid framework. In this regard, Bhattacharyya et al. [23] examined the nanofluid passing through a shrinking wall. Inspired by the knowledge gap, we have extended their work by adding aligned MHD and converting the Casson fluid into an engine oil-based Casson nanofluid with CNTs, power law index, wall mass exchange parameter, and thermal radiation past a shrinking wall through Kummer’s functions. It is noted that the temperature distribution of nanofluids can be controlled by the power law index parameter. Additionally, the stream lines and isotherms are plotted to visualize the flow pattern and temperature distribution.

## 3. Flow Analysis

To construct the model, an incompressible, steady, and 2D flow of an engine oil-based Casson nanofluid past a shrinking sheet occupying space y>0 is examined. The CNT is taken as nanoparticles. Consider the shrinking surface along *x*-axis with velocity $u=a_{s}x$. Additionally, the effects of heat radiation are also used together with the normally enforced magnetic field $B_{0}$ to the moving fluid. Additionally, we suppose that a Casson fluid’s isotropic, incompressible flow can be described by the following rheological equation of state [6,23]:

(1) $$\begin{array}{c} \tau_{ij}=\left\{\begin{array}{c} \left(2\mu_{cntf}\left(B_{v}\right)+\frac{P_{ye}}{\sqrt{2\pi }}\right)e_{ij},\;\;\;\mathrm{when}\;\pi >\pi_{cv},\\ \left(2\mu_{cntf}\left(B_{v}\right)+\frac{P_{ye}}{\sqrt{2\pi_{cv}}}\right)e_{ij},\;\mathrm{when}\;\pi <\pi_{cv}, \end{array}\right. \end{array}$$

where $\tau_{ij}=$ the stress tensor element, $e_{ij}=$ the strain tensor fraction, $P_{ye}=$ the fluid’s yield stress, $\mu_{cntf}\left(B_{v}\right)=$ plastic dynamic viscosity of non-Newtonian fluid, and $\pi =e_{ij}e_{ij}$ and $\pi_{cv}=$ the critical value dependant on the non-Newtonian fluid problem. The essential equations are as follows [6]:

(2) $$\begin{array}{cc} \; & \frac{\partial u}{\partial x}+\frac{\partial v}{\partial y}=0, \end{array}$$

(3) $$\begin{array}{cc} \; & u\frac{\partial u}{\partial x}+v\frac{\partial u}{\partial y}=\frac{\mu_{cntf}}{\rho_{cntf}}\left(1+\frac{1}{\kappa }\right)\frac{\partial^{2}u}{\partial y^{2}}-\frac{\sigma_{cntf}B_{0}^{2}\sin^{2}\omega }{\rho_{cntf}}u, \end{array}$$

the following are the suitable boundary conditions for the problem [23]:

(4) $$u=-U_{sw},\;v=-v_{sw}\;\mathrm{at}\;y=0;\;u=0,\;\mathrm{as}\;y\to \infty ,$$

where velocity of the fluid along *x* and *y* coordinate is expressed by *u* and *v*, respectively. $\mu_{cntf}$ is the dynamic viscosity, the Casson parameter $=\kappa =\mu_{cntf}\left(B_{v}\right)\sqrt{2\pi_{cv}}/P_{ye}$, $B_{0}$ the magnetic entity, $U_{sw}=a_{s}x$ is the surface shrinking velocity accompanying $a_{s}>0$, $v_{sw}=$ the wall mass transport entity (suction/injection), $\sigma_{cntf}$ is the electrical conductivity, and $\rho_{cntf}=$ the density. Here subscript cntf describes the nanofluid. The following are the characteristics of the nanofluid [24,25,26]:

(5) $$\left.\begin{array}{cc} \; & \alpha_{cntf}=\frac{k_{cntf}}{{\left(\rho c_{p}\right)}_{cntf}},\;\mu_{cntf}{\left(1-\Phi \right)}^{2.5}=\mu_{fe},\\ \; & {\left(\rho c_{p}\right)}_{cntf}=\Phi {\left(\rho c_{p}\right)}_{cntf}+\left(1-\Phi \right){\left(\rho c_{p}\right)}_{fe},\\ \; & \rho_{cntf}=\Phi \left(\rho_{cntf}\right)+\left(1-\Phi \right)\rho_{fe},\;\nu_{cntf}=\frac{\mu_{cntf}}{\rho_{cntf}},\\ \; & k_{cntf}=k_{fe}\frac{1-\Phi +2\;\frac{\Phi \;k_{\mathrm{cntf}}}{k_{\mathrm{cntf}}-k_{\mathrm{fe}}}\ln\left(1/2\;\frac{k_{\mathrm{cntf}}+k_{\mathrm{fe}}}{k_{\mathrm{fe}}}\right)}{1-\Phi +2\;\frac{\Phi \;k_{\mathrm{fe}}}{K_{\mathrm{cntf}}-k_{\mathrm{fe}}}\ln\left(1/2\;\frac{k_{\mathrm{cntf}}+k_{\mathrm{fe}}}{k_{\mathrm{fe}}}\right)},\\ \; & \sigma_{cntf}=\frac{3\Phi \sigma_{fe}\left(\frac{\sigma_{cntf}}{\sigma_{fe}}-1\right)}{-\Phi \left(\frac{\sigma_{cntf}}{\sigma_{fe}}-1\right)+\left(\frac{\sigma_{cntf}}{\sigma_{fe}}+2\right)}+\sigma_{fe}, \end{array}\right\}$$

where the thermal conductivity of the nanofluid $=k_{cntf}$ and the effective heat capacity, density, particle volume fraction, and the thermal conductivity of the engine oil are represented by ${\left(\rho c_{p}\right)}_{fe}$, $\rho_{fe}$, Φ, and $k_{fe}$, respectively. The following variables define similarity [24]:

(6) $$\begin{array}{cc} \; & u=a_{s}xf^{\prime }\left(\eta \right),\;\;v=-{\left(\nu_{fe}a_{s}\right)}^{1/2}f\left(\eta \right),\;\;\eta =y{\left(\frac{a_{s}}{\nu_{fc}}\right)}^{1/2},\;\;\theta \left(\eta \right)=\frac{T-T_{\infty }}{T_{w}-T_{\infty }}. \end{array}$$

Equation (2) is automatically satisfied as a result of the foregoing transformation, and (6), (3), and (4) are transformed to:

(7) $$\begin{array}{c} \left(1+\frac{1}{\kappa }\right)f^{\prime \prime \prime }-\beta_{1}\;\beta_{2}\;{f^{\prime }}^{2}-\beta_{1}\;M_{egt}\sin^{2}\omega f^{\prime }+\beta_{2}\;\beta_{1}\;ff^{\prime \prime }=0, \end{array}$$

(8) $$f\left(\eta \right)=\Omega ,\;f^{\prime }\left(\eta \right)=-1,\;\mathrm{at}\;\eta =0;\;f^{\prime }\left(\eta \right)\to 0\;\mathrm{as}\;\eta \to \infty .$$

Here, the suction/injection parameter $=\Omega =\frac{v_{sw}}{\sqrt{a_{s}\nu_{fe}}}$, $\beta_{1}={\left(1-\Phi \right)}^{2.5}$, $\beta_{2}=\left(\frac{\rho_{cntf}}{\rho_{fe}}\Phi -\Phi +1\right)$, and $M_{egt}=\frac{\sigma_{fe}B_{0}^{2}}{a_{s}\rho_{fe}}$ the Hartmann number. The solution of (7) in closed form is as follows [27]:

(9) $$\begin{array}{cc} \; & f\left(\eta \right)=\Lambda_{1}+\Lambda_{2}e^{-\Psi \eta }, \end{array}$$

where $\Lambda_{1}$, $\Lambda_{2}$, and Ψ are constant with Ψ>0. Using (8), determine the answer to (7) as shown below

(10) $$\begin{array}{cc} \; & f\left(\eta \right)=\Omega -\frac{1}{\Psi }+\frac{e^{-\Psi \eta }}{\Psi }. \end{array}$$

Using (7) and (10) together, we get following

$$\begin{array}{cc} \Psi = & \;\frac{\sqrt{{\beta_{1}}^{2}{\beta_{2}}^{2}\kappa^{2}\Omega^{2}+4\;\beta_{1}\;\kappa^{2}M_{egt}\sin^{2}\omega +4\;M_{egt}\beta_{1}\kappa \sin^{2}\omega -4\;\beta_{1}\;\beta_{2}\;\kappa^{2}-4\;\beta_{1}\;\beta_{2}\;\kappa }}{\kappa +1}\\ & +\frac{\beta_{1}\;\beta_{2}\;\kappa \Omega }{2(\kappa +1)}. \end{array}$$

## 4. Heat Transfer Analysis

The heat transfer examination with the impact of the thermal radiation phenomena is presented in this part. The basic equation is as follows [28]:

(11) $$\begin{array}{cc} \; & u\frac{\partial T}{\partial x}+v\frac{\partial T}{\partial y}=\alpha_{cntf}\frac{\partial^{2}T}{\partial y^{2}}-\frac{1}{{\left(\rho c_{p}\right)}_{cntf}}\frac{\partial Q_{rd}}{\partial y}, \end{array}$$

where $Q_{rd}=-\frac{\sigma^{*}}{3k^{*}}\frac{\partial T^{4}}{\partial y}$ and T= the temperature of sheet. After expanding Taylor’s series of $T^{4}$ by around $T_{\infty }$ and ignoring higher order terms, we obtain the following equation:

(12) $$\begin{array}{cc} \; & Q_{rd}=-\frac{2^{4}\sigma^{*}}{3k^{*}}T_{\infty }^{3}\frac{\partial T}{\partial y}, \end{array}$$

where $\sigma^{*}$ is Stefan’s constant and the mass absorption coefficient $=k^{*}$. Converting (13) into (11), (11) becomes as follows:

(13) $$\begin{array}{cc} \; & u\frac{\partial T}{\partial x}+v\frac{\partial T}{\partial y}=\alpha_{cntf}\frac{\partial^{2}T}{\partial y^{2}}-\frac{2^{4}\sigma^{*}}{3k^{*}}T_{\infty }^{3}\frac{\partial T}{\partial y}, \end{array}$$

the necessary boundary constraints:

(14) $$T=T_{\infty }+T_{0f}x^{N}\;\mathrm{at}\;y=0;\;T\to T_{\infty }\;\mathrm{as}\;y\to \infty .$$

Here, the specific heat shows by ${\left(c_{p}\right)}_{cntf}$, $T_{0f}$ is a constant that relies over the fluid’s thermal characteristics, thermal diffusivity $=\alpha_{cntf}$, *N* the power law index, and $T_{\infty }=$ the free stream temperature. The following energy expression is obtained after utilizing (5) and (13) together:

(15) $$\begin{array}{c} \lambda \theta_{\eta \eta }-\left(Nf_{\eta }\theta -f\theta_{\eta }\right)Pr=0, \end{array}$$

where

(16) $$\begin{array}{cc} & \beta_{3}=\frac{\left(k_{cntf}+2k_{fe}\right)-2\Phi \left(k_{fe}-k_{cntf}\right)}{\left(k_{cntf}+2k_{fe}\right)+2\Phi \left(k_{fe}-k_{cntf}\right)},\;\beta_{4}=\left(1-\Phi +\Phi \frac{{\left(\rho cp\right)}_{cntf}}{{\left(\rho cp\right)}_{fe}}\right)\\ \; & Pr=\frac{\nu_{fe}}{\alpha_{fe}},\;\Upsilon =\frac{k^{*}k_{fe}}{2^{2}\sigma^{*}T_{\infty }^{3}},\;\lambda =\left(\frac{\beta_{3}}{\beta_{4}}\frac{3\Upsilon \beta_{3}+4}{3\Upsilon \beta_{3}}\right). \end{array}$$

Here, the radiation entity =Υ and Pr= the Prandtl number. The changed boundary restrictions are:

(17) $$\left.\begin{array}{cc} \; & \theta (\eta )=1\;\;\;\;\mathrm{at}\;\;\eta =0,\\ \; & \theta (\eta )\to 0\;\;\;\;\;\mathrm{as}\;\;\eta \to \infty . \end{array}\right\},$$

As a result, it is easily obtained by inputting (10) into (15) as:

(18) $$\begin{array}{c} \lambda \theta_{\eta \eta }-\left(N\left(-e^{-\Psi \eta }\right)\theta -\left(\Omega -\frac{1}{\Psi }+\frac{e^{-\Psi \eta }}{\Psi }\right)\theta_{\eta }\right)Pr=0, \end{array}$$

currently, a further variable

(19) $$\begin{array}{c} \varsigma =\frac{Pr\;e^{-\Psi \eta }}{\lambda \Psi^{2}}, \end{array}$$

is presented. Therefore, the Equation (18) becomes:

(20) $$\begin{array}{cc} \; & \varsigma \frac{\partial^{2}\theta }{\partial \varsigma^{2}}+\left(\psi -\varsigma \right)\frac{\partial \theta }{\partial \varsigma }+N\theta =0, \end{array}$$

where $\psi =(1-\psi_{1})$ and $\psi_{1}=\frac{Pr}{\lambda \Psi }\left(\Omega -\frac{1}{\Psi }\right)$. The following are the amended boundary criteria:

(21) $$\begin{array}{cc} \; & \theta (\varsigma )=1,\;\;\;\;\;\theta (0)=0. \end{array}$$

The closed type solution of (20) with (21) is, with regard to Kummer’s functions [29]:

(22) $$\begin{array}{c} \theta \left(\varsigma \right)={\frac{\lambda \Psi^{2}\varsigma }{Pr}}^{-\frac{\mathrm{Pr}\;}{\lambda }\left(\Omega -\frac{1}{\Psi }\right)}\frac{M\left(\frac{\mathrm{Pr}}{\lambda \;\Psi }\left(\Omega -\Psi^{-1}\right)-N,1+\frac{\mathrm{Pr}}{\lambda \;\Psi }\left(\Omega -\Psi^{-1}\right),\varsigma \right)}{M\left(\frac{\mathrm{Pr}}{\lambda \;\Psi }\left(\Omega -\Psi^{-1}\right)-N,1+\frac{\mathrm{Pr}}{\lambda \;\Psi }\left(\Omega -\Psi^{-1}\right),\frac{\mathrm{Pr}}{\lambda \;\Psi^{2}}\right)}, \end{array}$$

we acquired the following solution:

(23) $$\begin{array}{c} \theta \left(\eta \right)=e^{-\frac{\mathrm{Pr}\;\eta }{\lambda }\left(\Omega -\frac{1}{\Psi }\right)}\frac{M\left(\frac{\mathrm{Pr}}{\lambda \;\Psi }\left(\Omega -\Psi^{-1}\right)-N,1+\frac{\mathrm{Pr}}{\lambda \;\Psi }\left(\Omega -\Psi^{-1}\right),\frac{\mathrm{Pr}\;e^{-\Psi \eta }}{\lambda \;\Psi^{2}}\right)}{M\left(\frac{\mathrm{Pr}}{\lambda \;\Psi }\left(\Omega -\Psi^{-1}\right)-N,1+\frac{\mathrm{Pr}}{\lambda \;\Psi }\left(\Omega -\Psi^{-1}\right),\frac{\mathrm{Pr}}{\lambda \;\Psi^{2}}\right)}, \end{array}$$

where M is the confluent hypergeometric function in this scenario.

## 5. Skin Friction and Local Nusselt Number

The local skin friction is calculated as:

(24) $$\begin{array}{cc} \; & C_{f}=\frac{\tau_{sw}}{\rho u_{sw}^{2}}=\beta_{1}C_{f}Re_{x}^{-1/2}=\left(1+\frac{1}{\kappa }\right)f^{\prime \prime }\left(0\right), \end{array}$$

where

$$\begin{array}{cc} & \tau_{sw}=\mu_{cntf}{\left(\frac{\partial u}{\partial y}\right)}_{y=0}=\mathrm{the}\;\mathrm{stress}\;\mathrm{at}\;\mathrm{wall},\\ & Re_{x}=\frac{xu_{sw}}{\nu }=\mathrm{the}\;\mathrm{Reynolds}\;\mathrm{number}. \end{array}$$

The gradient of temperature at the wall is expressed as:

(25) $$\begin{array}{cc} \; & Nu=\frac{-k_{cntf}x}{k_{fe}\left(T_{w}-T_{\infty }\right)}{\left(\frac{\partial T}{\partial y}\right)}_{y=0}=\frac{k_{fe}}{k_{cntf}}Nu_{x}Re_{x}^{-1/2}=-\theta_{\eta }\left(0\right). \end{array}$$

## 6. Results and Discussion

**Figure 1:**
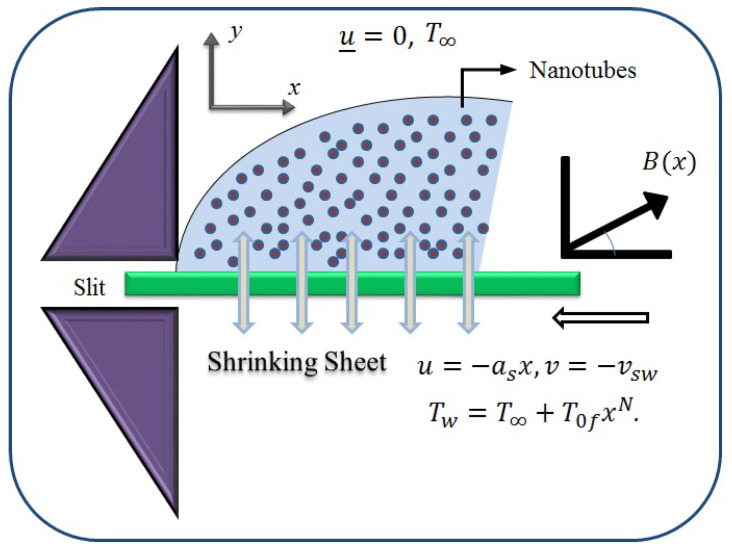
The physical viewpoint of the concept.

**Figure 2:**
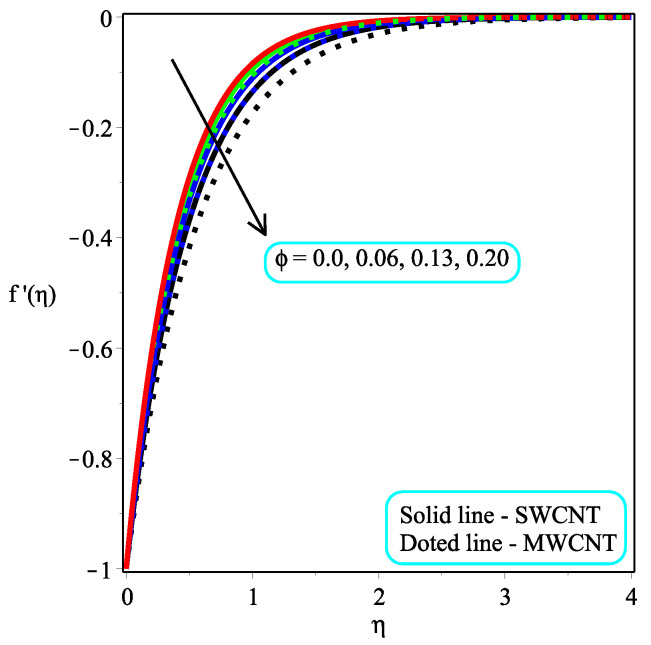
Repercussion of ϕ with $M_{egt}=1$, Ω=3, κ=2, ω=π/3 on velocity on velocity profiles.

**Figure 3:**
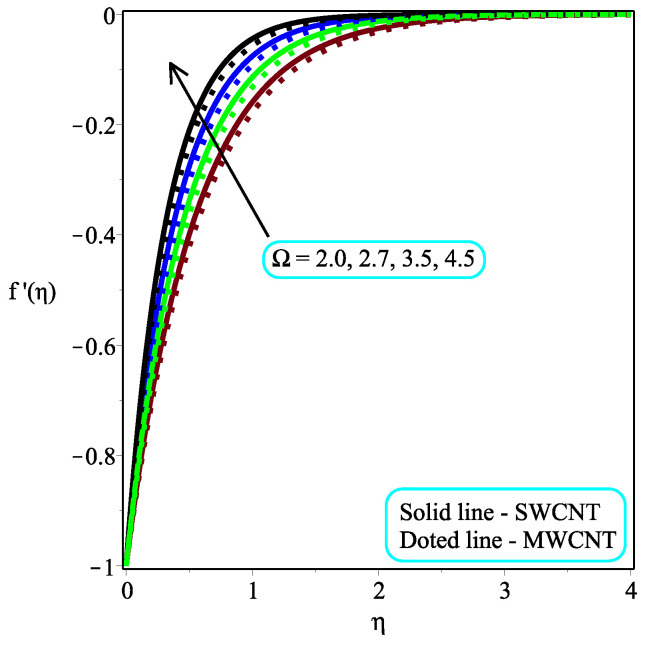
Repercussion of Ω with Φ=0.1, $M_{egt}=2$, κ=2, ω=π/4 on velocity profiles.

**Figure 4:**
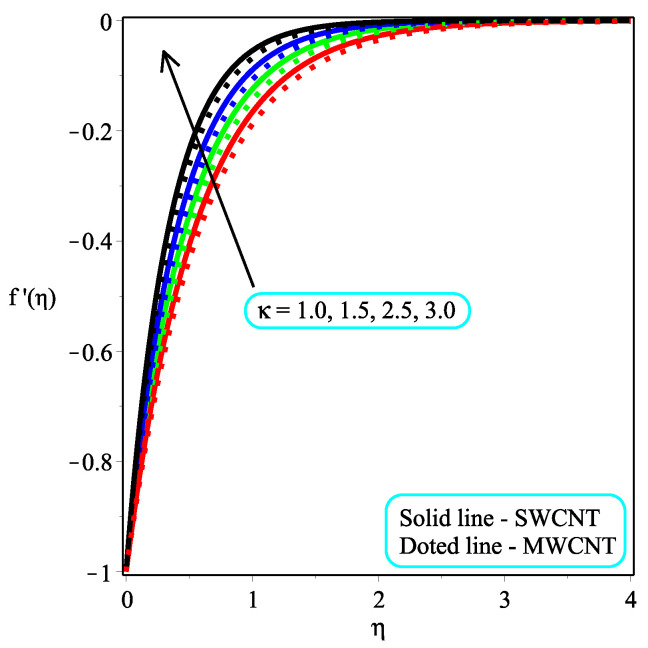
Repercussion of κ with Φ=0.1, $M_{egt}=2$, Ω=2, ω=π/4 on velocity profiles.

This part seeks to anticipate and visually characterize the performance of a Casson nanofluid based on CNT engine oil under the influence of significantly associated factors such as the velocity, temperature, skin friction coefficient, and local Nusselt number. The thermo-physical characteristics of engine oil and nanoparticles are shown in Table 1. Table 2 and Table 3 show the numerical measured values of skin friction and temperature gradient at the wall. Table 4 shows the comparison of the current study with existing results. The physical idea of the considered model is displayed in Figure 1. The consequence of ϕ on the dimensionless velocity distribution are visualized in Figure 2. It is worth noting that the velocity field decelerates with a rising value of ϕ. Furthermore, the velocity pattern of multi-walled CNTs drops significantly when compared to single-walled CNTs. Physically, the viscosity of the base fluid increases by adding the CNTs, and friction between the molecules also enhances, which leads to an enhancement in the velocity field. Figure 3 depicts the impact of Ω on $f^{\prime }\left(\eta \right)$. It is recognized that the magnitude of the $f^{\prime }\left(\eta \right)$ accelerates due to gaining the magnitude of Ω. Moreover, the gaining rate is higher in single-walled CNTs than in multi-walled CNTs. The impact of κ on $f^{\prime }\left(\eta \right)$ is examined in Figure 4. The dimensionless velocity field is dropped because of a decrement in κ. Physically, the output stress $P_{ye}$ decreases as the Casson parameter κ increases, and as a result, the thickness of the velocity boundary layer also declines.

**Figure 5:**
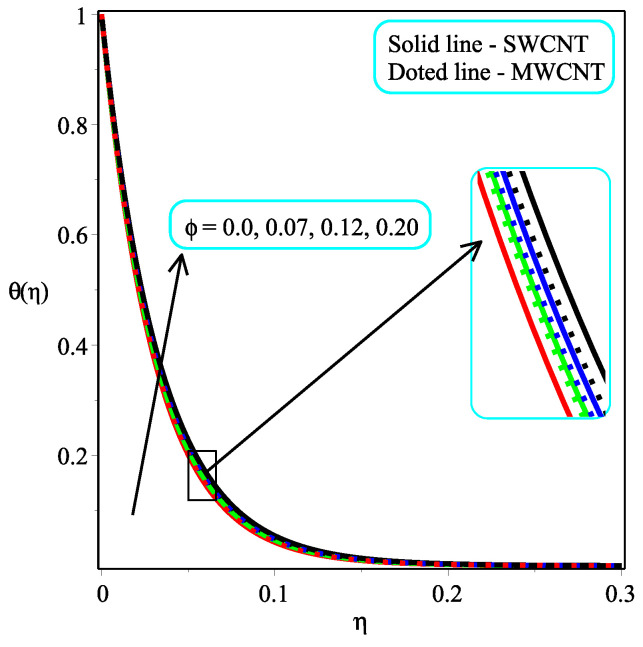
Repercussion of ϕ with κ=1$M_{egt}=2$, Ω=1.5, ω=π/3, N=2, Υ=0.4, Pr=100 on the temperature profile.

**Figure 6:**
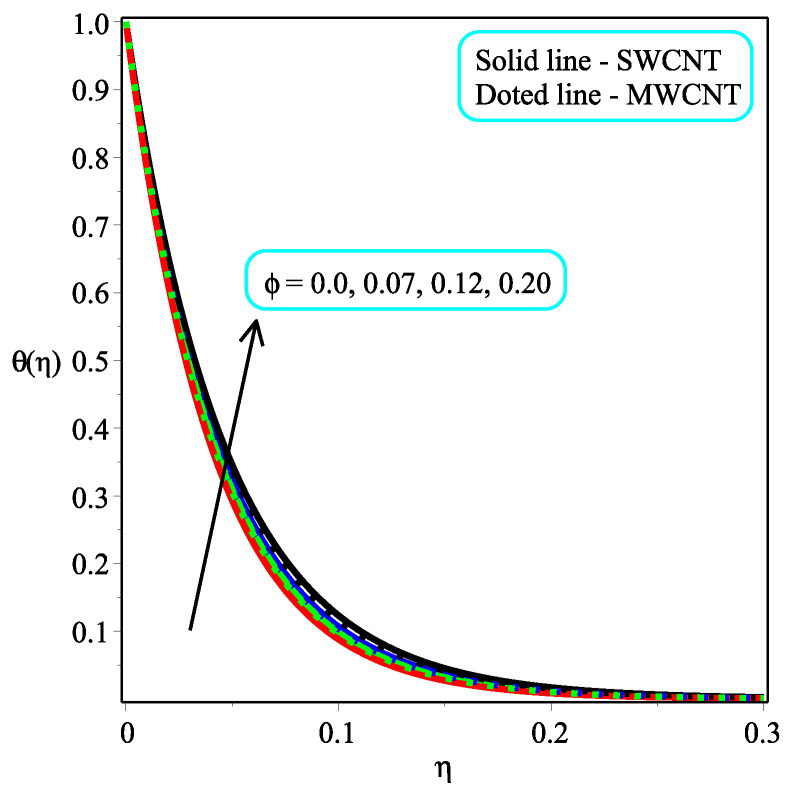
Repercussion of ϕ with κ=1$M_{egt}=2$, Ω=1.5, ω=π/3, N=10, Υ=0.4, Pr=100 on the temperature profile.

**Figure 7:**
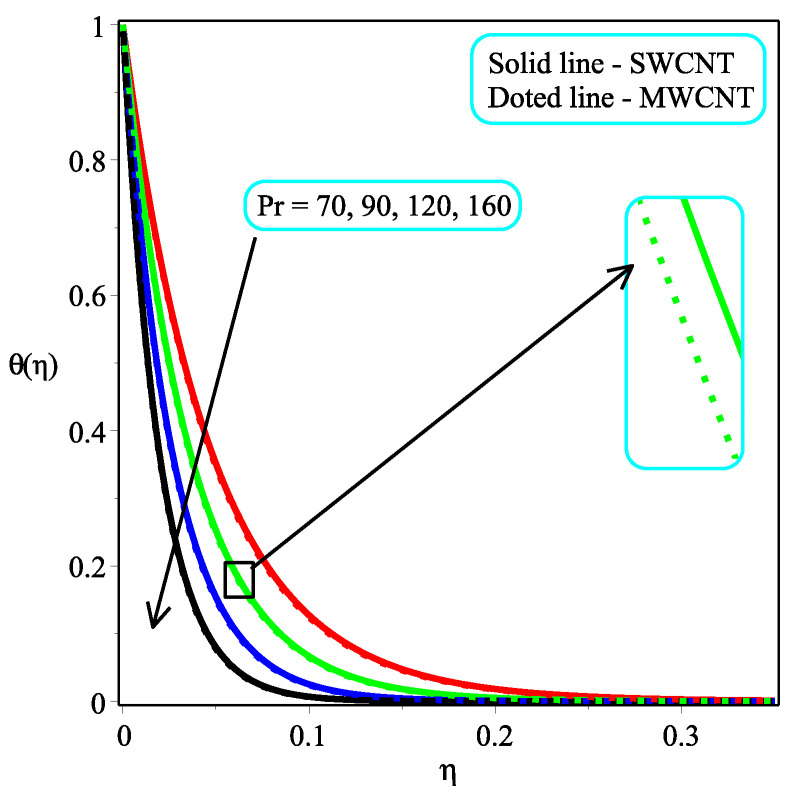
Repercussion of Pr with κ=1$M_{egt}=2$, Ω=1.5, ω=π/3, N=10, Υ=0.4, ϕ=0.1 on the temperature profile.

**Figure 8:**
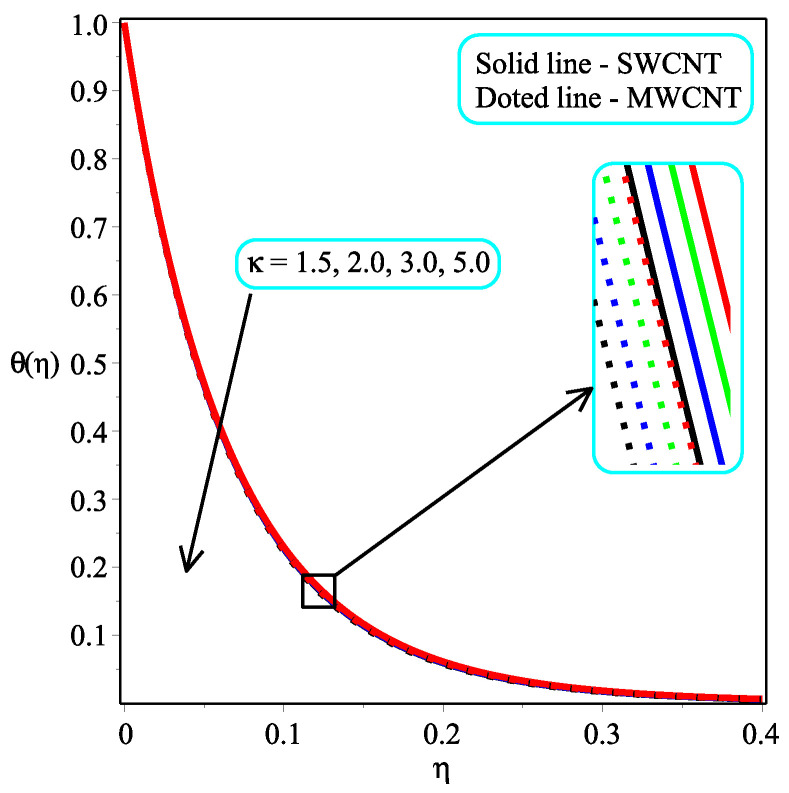
Repercussion of κ with ϕ=0.1$M_{egt}=2$, Ω=1, ω=π/3, N=1, Υ=0.3, Pr=100 on the temperature profile.

Figure 5 and Figure 6 portray the influence ϕ with N=2,10 on temperature distribution. It is noted that the amount of θ(η) enhances by accelerating the value of ϕ for both values of *N*. It is also seen that the rate of increase is more in the case of single-walled CNTs than in multi-walled CNTs. Physically, the fluid becomes denser as the concentration of nanoparticles rises. An increase in the volume percentage of nanoparticles improves the heat transmission of nanofluids, which raises the temperature. The consequence of Pr with Ω=1.5 over the temperature field is shown in Figure 7. It is perceived that the amount of θ(η) is decelerated with an increment in the magnitude of Pr. Physically, the proportion of momentum to thermal diffusivity is known as the Prandtl numberb (Pr). Pr regulates the comparative thickness of the momentum and thermal boundary layers in heat exchange issues. The decreasing ratio is slower in the case of single-walled CNTs than in multi-walled CNTs. In Figure 8, the effect of κ on the temperature distribution are presented. As the magnitude of κ increases, so the temperature distribution is decreased in both kinds of CNTs. In reality, increasing the Casson parameter κ prevents liquid motion by decreasing the yield stress of Casson fluid and increasing plastic dynamic viscosity.

**Figure 9:**
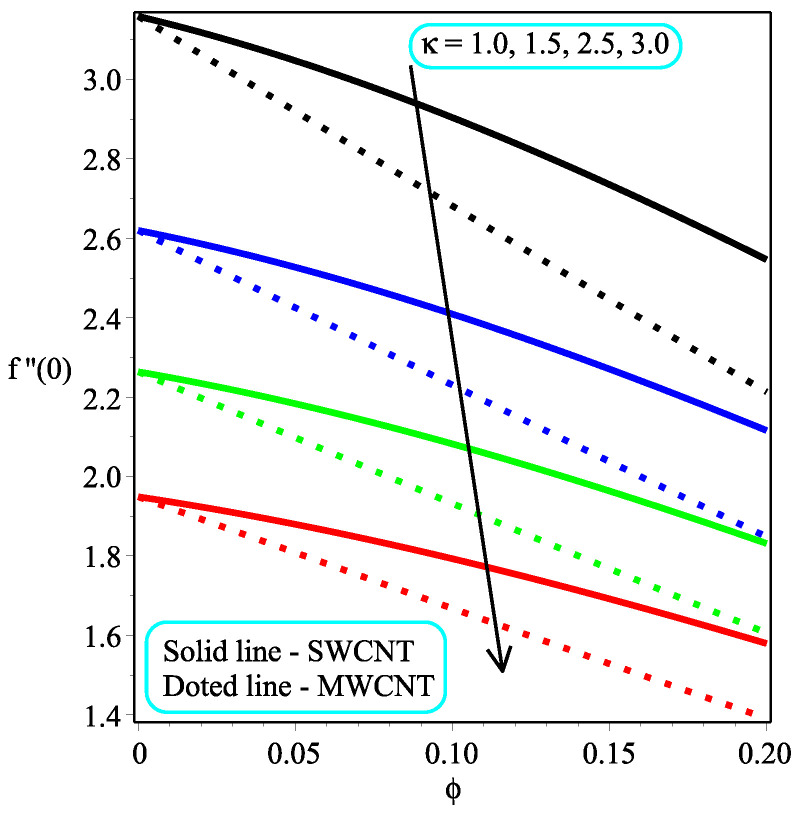
Repercussion of κ with $M_{egt}=2$, Ω=2, ω=π/4 on the local skin friction field.

**Figure 10:**
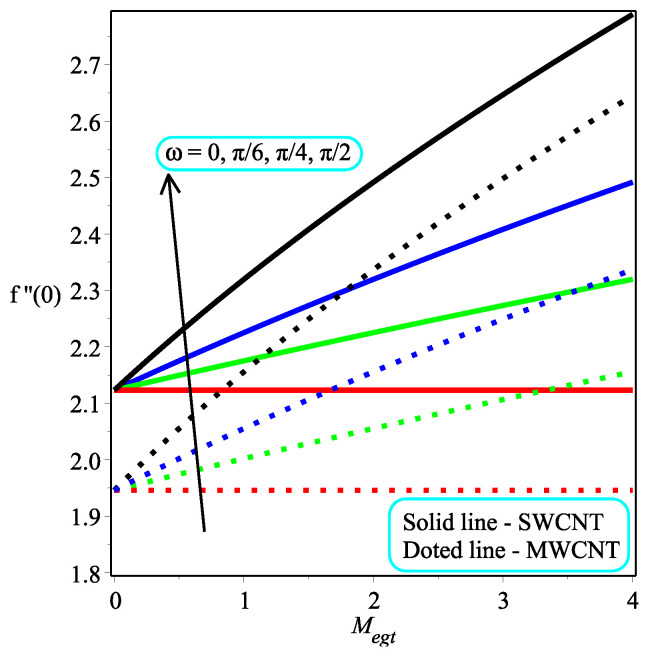
Repercussion of ω with κ=2, Ω=3, ϕ=0.1 on the local skin friction field.

**Figure 11:**
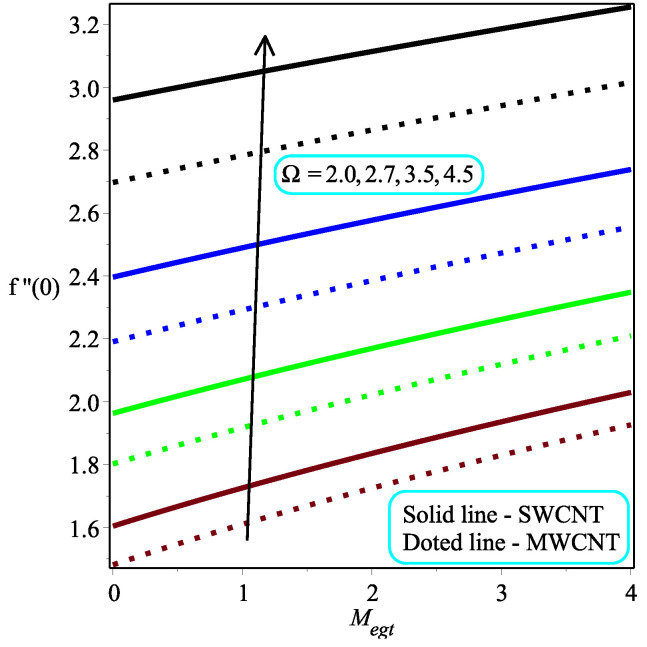
Repercussion of Ω with κ=2, ω=π/4, ϕ=0.1 on the local skin friction field.

**Figure 12:**
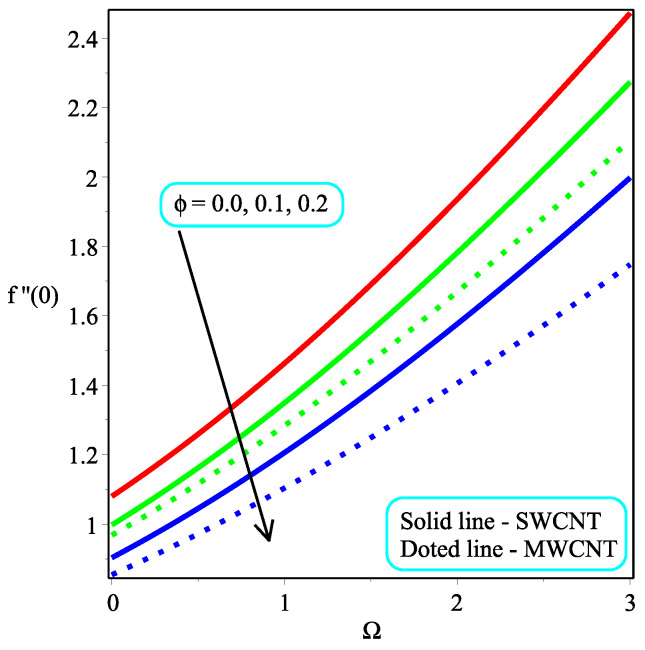
Repercussion of ϕ with κ=2, ω=π/3, $M_{egt}=1$ on the local skin friction field.

The impression of κ while varying the values of ϕ on the skin friction at the wall is depicted in Figure 9. The fact is that an increment in κ leads to a drop in the magnitude of skin friction at the wall. Physically, the applied stress tends to decrease as the magnitude of κ rises, resulting in a reduction in the coefficient of skin friction. Further, the skin friction at the wall rate is dropped in the multi-walled CNTs rapidly as compared to the single-walled CNTs. Figure 10 expresses the consequence of ω over the skin friction at the wall. The resistive forces are increased when enhancing the value of ω for which the drag force is increased, which leads to a decrease in the skin friction at the wall. It is also discovered that $M_{egt}$ has no effect at ω=0, and the minor effect of ω is investigated in the flow field. The local skin friction ratio is higher in the case of single-walled CNTs than in multi-walled CNTs. Figure 11 and Figure 12 show the impact of Ω and ϕ on $-f^{\prime \prime }\left(0\right)$, respectively. The local skin friction decelerates due to an increment in Ω and the opposite trend is shown in the case of ϕ for both of the CNTs.

**Figure 13:**
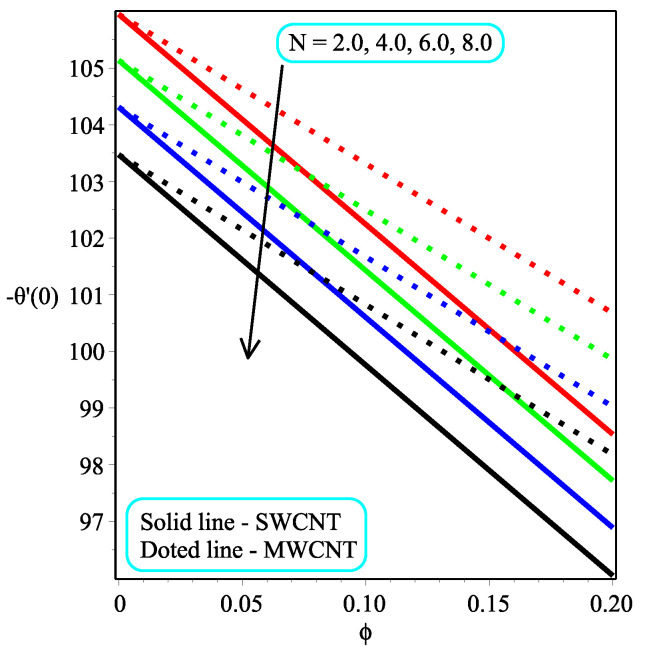
Repercussion of *N* with κ=1$M_{egt}=2$, Ω=2.5, ω=π/3, Υ=1, Pr=100 on −$\theta^{\prime }\left(0\right)$.

**Figure 14:**
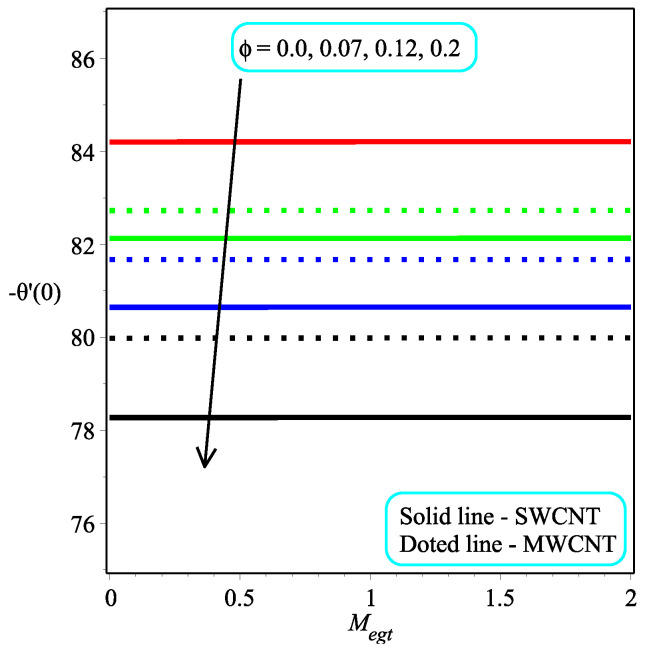
Repercussion of ϕ with κ=1, Ω=2, ω=π/3, N=10, Υ=1, Pr=100 on −$\theta^{\prime }\left(0\right)$.

**Figure 15:**
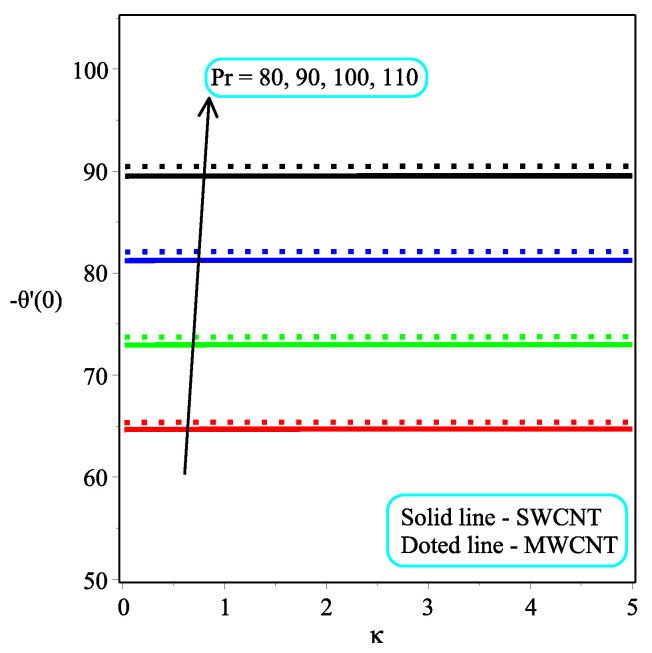
Repercussion of Pr with ϕ=0.1, $M_{egt}=2$, Ω=2, ω=π/3, N=2, Υ=1 on −$\theta^{\prime }\left(0\right)$.

**Figure 16:**
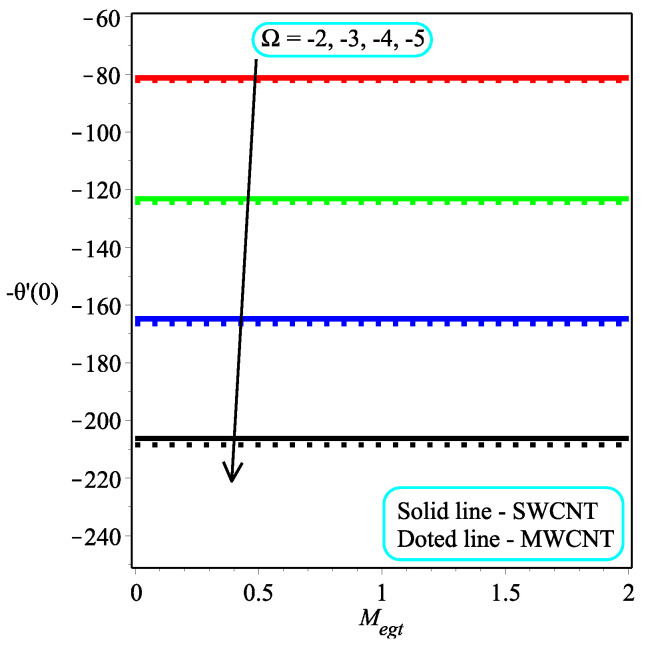
Repercussion of Ω with κ=1ϕ=0.1, ω=π/3, N=2, Υ=1, Pr=100 on −$\theta^{\prime }\left(0\right)$.

**Figure 17:**
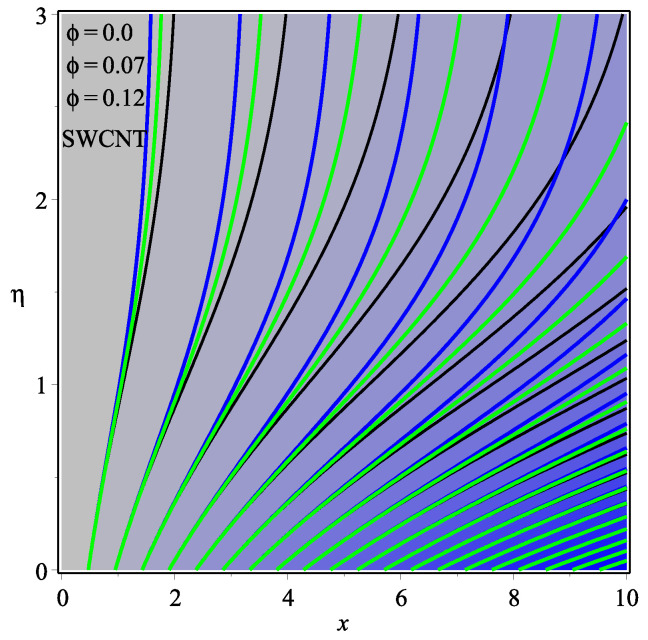
Repercussion of ϕ with $M_{egt}=0.5$, Ω=1, κ=2.5, ω=π/3 on stream lines.

**Figure 18:**
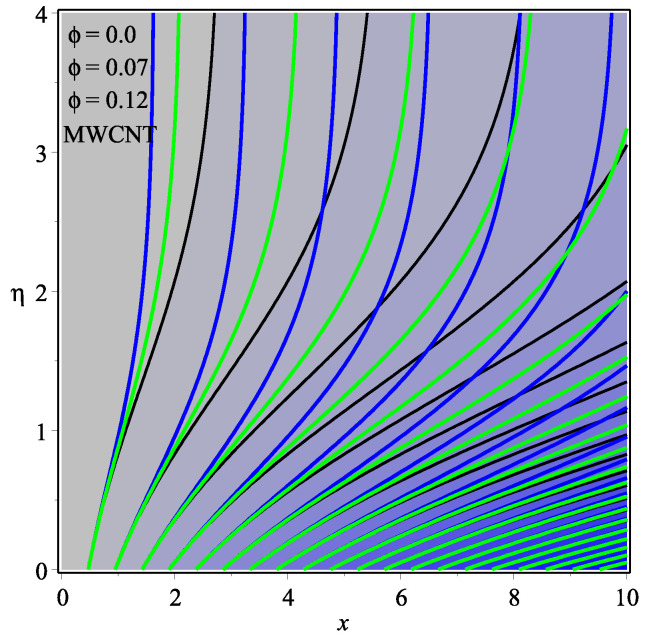
Repercussion of ϕ with $M_{egt}=0.5$, Ω=1, κ=2.5, ω=π/3 on stream lines.

**Figure 19:**
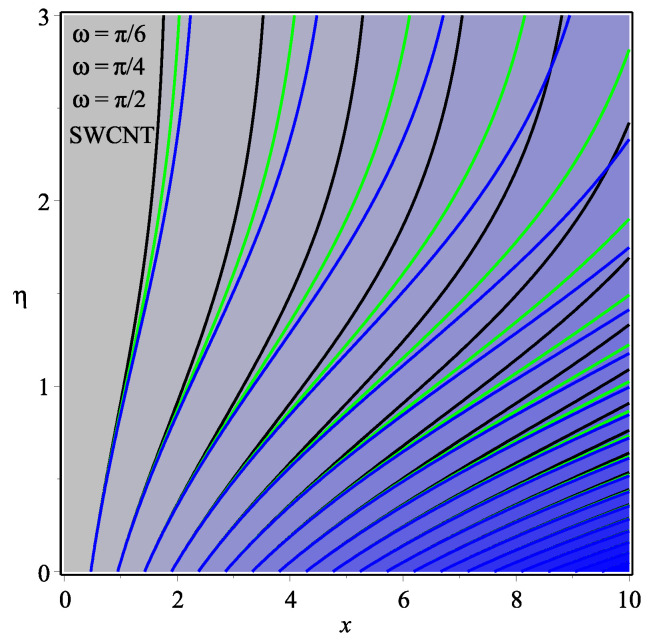
Repercussion of ω with ϕ=0.1, $M_{egt}=0.5$, Ω=1, κ=2.5 on stream lines.

**Figure 20:**
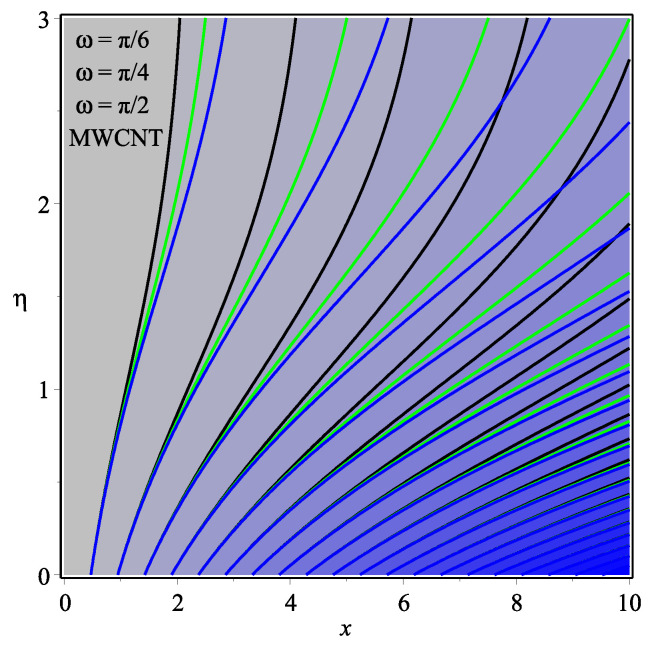
Repercussion of ω with ϕ=0.1, $M_{egt}=0.5$, Ω=1, κ=2.5 on stream lines.

**Figure 21:**
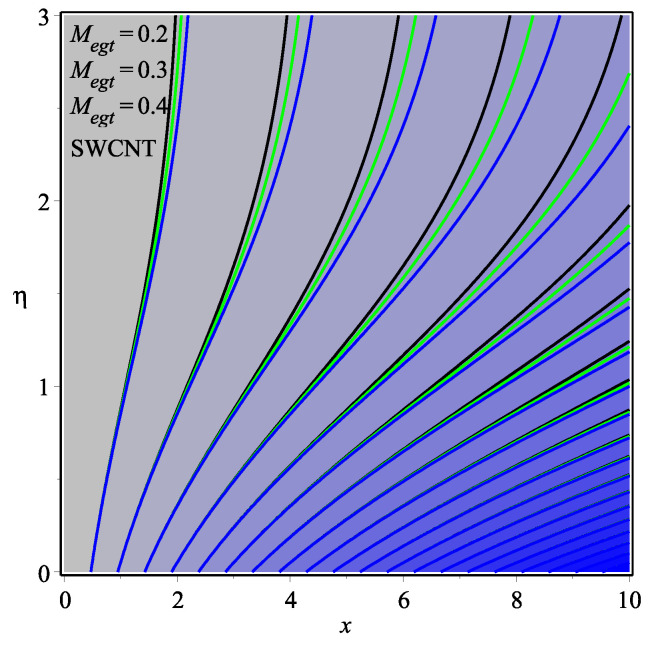
Repercussion of $M_{egt}$ with ϕ=0.1, ω=π/3, Ω=1, κ=2.5 on stream lines.

**Figure 22:**
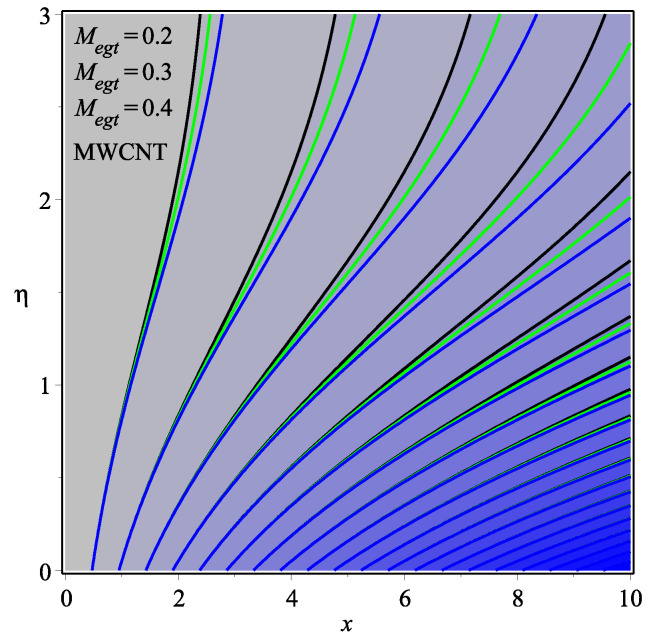
Repercussion of $M_{egt}$ with ϕ=0.1, ω=π/3, Ω=1, κ=2.5 on stream lines.

**Figure 23:**
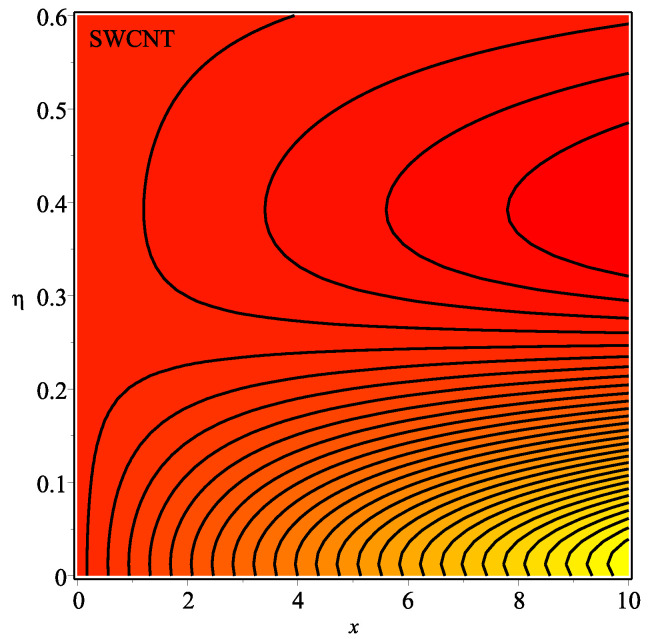
Repercussion of $M_{egt}=1$ with ϕ=0.1, κ=2, Ω=1, ω=π/2, N=10, Υ=0.2, Pr=100 on the isotherm.

**Figure 24:**
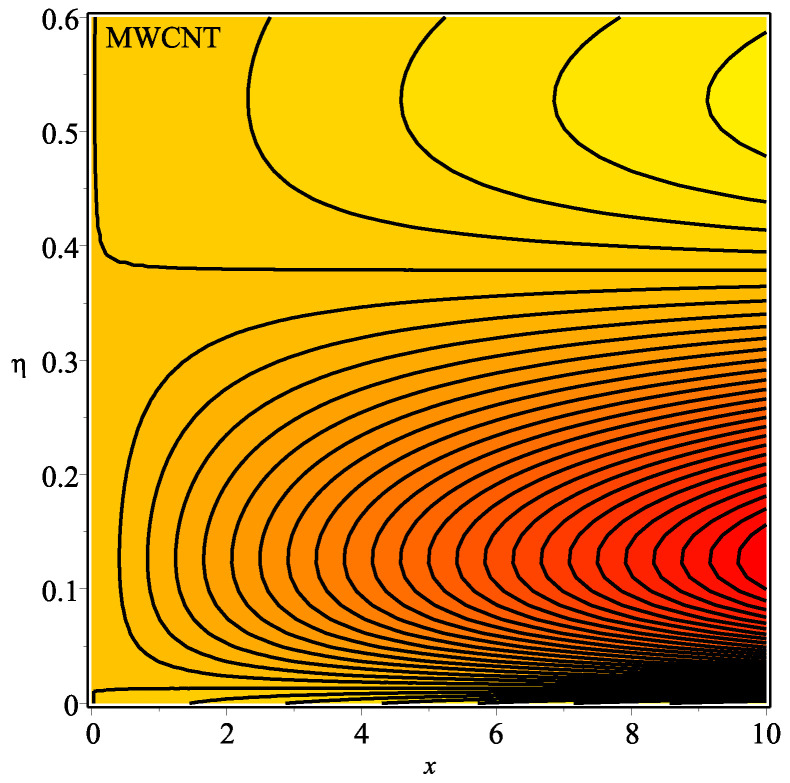
Repercussion of $M_{egt}=1$ with ϕ=0.1, κ=2, Ω=1, ω=π/2, N=10, Υ=0.2, Pr=100 on the isotherm.

**Figure 25:**
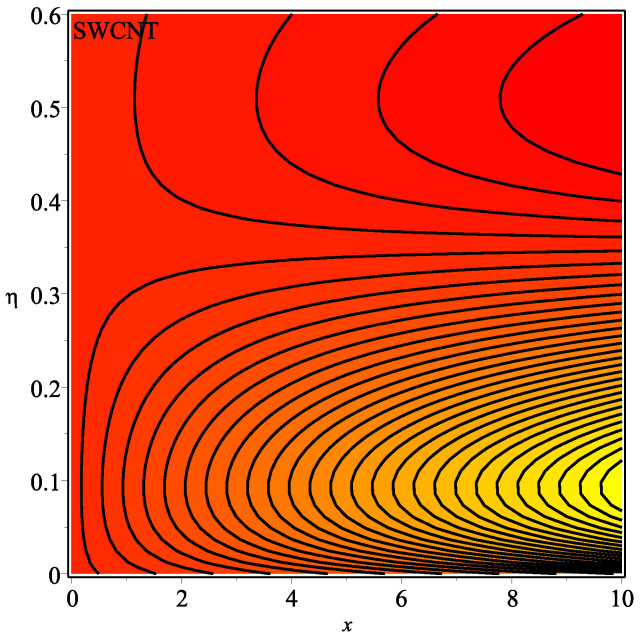
Repercussion of $M_{egt}=2$ with ϕ=0.1, κ=2, Ω=1, ω=π/2, N=10, Υ=0.2, Pr=100 on the isotherm.

**Figure 26:**
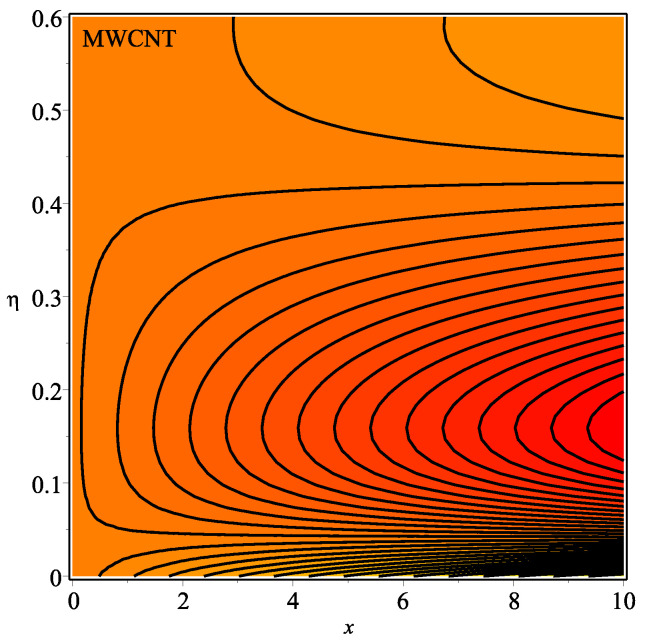
Repercussion of $M_{egt}=2$ with ϕ=0.1, κ=2, Ω=1, ω=π/2, N=10, Υ=0.2, Pr=100 on the isotherm.

Figure 13 shows the influence of *N* on the temperature gradient at wall with $M_{egt}$. A significant decrease is observed with the enhancing values of $M_{egt}$ in the case of single-walled CNTs and multi-walled CNTs. Physically, the conductive energy exchange is higher than convective heat transport, which leads to a decrement in the Nusselt number. The variation of ϕ on the temperature gradient at the wall is examined in Figure 14. A decreasing trend is observed due to an increment in ϕ. Figure 15 and Figure 16 depict the effect of Pr and Ω on $-\theta^{\prime }\left(0\right)$, respectively. The magnitude of $-\theta^{\prime }\left(0\right)$ diminishes as we enhance the amount of Pr and the opposite trend is shown for Ω. Physically, Pr is the ratio of momentum exchange to heat transport; actually, an increase in Pr is due to a decrease in heat transport, which leads to a decrease in the Nusselt number. Figure 17 and Figure 18 are plotted to visualize the stream lines with different values of ϕ for both single-walled CNTs and multi-walled CNTs. It is seen that the stream lines are thicker in the case of single-walled CNT-based fluid as compared to multi-walled CNT-based fluids. The impact of ω on stream line is investigated in Figure 19 and Figure 20 for both single-walled CNTs and multi-walled CNTs. It is shown that the stream lines are thicker in the case of single-walled CNT-based fluid as compared to multi-walled CNT-based fluid. The same behavior is observed for $M_{egt}$ in Figure 21 and Figure 22. Figure 23 and Figure 24 describes the impact of $M_{egt}$ on isotherm with $M_{egt}=1$ for single-walled CNT-based fluid and multi-walled CNT-based fluid, respectively. The influence of $M_{egt}$ on isotherm with $M_{egt}=2$ for ingle-walled CNT-based fluid and multi-walled CNT-based fluid is observed in Figure 25 and Figure 26, respectively. It is noted that more temperature distribution occurs in the case of multi-walled CNT-based fluid as compared to single-walled CNT-based fluid.

## 7. Conclusions

The aligned MHD engine oil-based Casson nanofluid with carbon nanotubes (single and multi-wall) passed a shrinking sheet accompanying the thermal radiation wall mass transport phenomena. The following findings are achieved:

- When $\omega =0^{\circ }$, the magnetic field has no effect on the velocity distribution, but it behaves transversely when ω=π/2 across the stream portion.
- The temperature distribution can be controlled through the power law index *N*.
- An increase in κ and ω leads to a decline in the value of skin friction at the wall, and the skin friction at the wall rate is dropped in the multi-walled CNTs rapidly as compared to the single-walled CNTs.
- The injection parameter decreases the heat transfer rate of the sheet. The single-walled CNTs have a less degradation rate as compared to multi-walled CNTs.
- It is examined that higher temperature distribution occurs in the case of a multi-walled CNT-based fluid as compared to a single-walled CNT-based fluid.

## Figures and Tables

**Table 1:** Thermal properties of engine oil and CNTs (SWCNT/MWCNT) [30,31,32].

Item	Name	$\frac{\rho }{\mathrm{kg}/m^{3}}$	$\frac{c_{p}}{j/\mathrm{kg}}$	$\frac{k}{W/m}$
Host Fluid	Engine oil	884	1910	0.144
	SWCNT	2600	425	6600
Nanoparticles	MWCNT	1600	796	3000

**Table 2:** Numerical table of $-f^{\prime \prime }\left(0\right)$ with κ=2 and $\omega =\frac{\pi }{4}$.

ϕ	$M_{\mathrm{egt}}$	Ω	SWCNT	MWCNT
0	0.2	3	2.316561177	2.316561177
0.09			2.165881457	2.003404391
0.14			2.050451725	1.830416322
0.20			1.889865256	1.626434980
0.1	0.2	3	2.144364785	1.968666416
	0.4		2.165069733	1.990956551
	0.6		2.185436595	2.012826468
	0.8		2.205481411	2.034299073
0.1	0.2	2.5	1.881661396	2.034299073
		3.5	2.415525397	2.211777817
		4.5	2.975599453	2.714870113
		5.5	3.551219031	3.232940895

**Table 3:** The value table of $-\theta^{\prime }\left(0\right)$ with κ=1, $\omega =\frac{\pi }{3}$ and Pr=100.

$M_{\mathrm{egt}}$	ϕ	*N*	Υ	SWCNT	MWCNT
0.2	0.1	2	1	62.46294101	65.67768785
0.4				62.46340335	65.67816046
0.6				62.46385308	65.67861805
0.8				62.46429066	65.67906178
0.2	0	2	1	127.5753320	127.5753320
	0.09			66.30454502	69.47237912
	0.14			49.96946560	53.20004770
	0.20			36.98735855	39.99738167
0.2	0.1	1	1	62.79765517	66.01305649
		2		62.46294101	65.67768785
		3		62.12468837	65.33892386
		4		61.78278446	64.99665998
0.2	0.1	2	1	62.46294101	65.67768785
			2	48.11850998	50.14347211
			3	39.06492988	40.48097101
			4	32.83111149	33.89048821

**Table 4:** The values of $f^{\prime \prime }\left(0\right)$ with ϕ=0 and $M_{egt}=0$.

κ	Ω	Bhattacharyya et al. [23]	Present Results
0.5	3.5	0.66667	0.666666666
1	4	1.70711	1.707106781
2	4.5	2.75831	2.758305739
5	5	3.95602	3.956017087

## Data Availability

All the data available inside the research work.

## Nomenclature

$B_{0}$	Magnetic field (Nm/A)
$\tau_{ij}$	Stress tensor element
$e_{ij}$	Strain tensor fraction
$P_{ye}$	Fluid’s yield stress
cntf	Subscript for nanofluid
$\mu_{cntf}\left(B_{v}\right)$	Plastic dynamic viscosity of non-Newtonian fluid
π, $\pi_{cv}$	Critical values
*x*, *y*	Coordinates
u,v	Velocity components (m/s)
κ	Casson parameter
$U_{sw}$	Surface shrinking velocity (m/s)
$v_{sw}$	Wall mass transport parameter
$\sigma_{cntf}$	Electrical conductivity of nanofluid (S/m)
$\rho_{cntf}$	Nanofluid density (Kg/$m^{3}$)
cntf	Carbon nanotube fluid
$k_{cntf}$	Thermal conductivity of nanofluid (W/mK)
${\left(c_{p}\right)}_{fe}$	Specific heat capacity (J/KgK)
$\rho_{fe}$	Density of base fluid (Kg/$m^{3})$
$k_{fe}$	Thermal conductivity of base fluid (W/mK)
Φ	Solid volume fraction
Ω	Suction/injection parameter
$M_{egt}$	Hartman number
$\Lambda_{1}$, $\Lambda_{2}$, Ψ	Constants
${\left(c_{p}\right)}_{cntf}$	Specific heat capacity of nanofluid (J/KgK)
Υ	Radiation parameter
*N*	Power law index
$Q_{rd}$	Radiative heat flux
*T*	Temperature (K)
$T_{0f}$	Sheet temperature (K)
$T_{\infty }$	Free stream temperature (K)
$\sigma^{*}$	Stefan’s constant
$k^{*}$	Mass absorption coefficient
$\alpha_{cntf}$	Thermal diffusivity $(m^{2}$/s)
Pr	Prandtl number
*M*	Confluent hyper-geometric function
$C_{f}$	Skin friction coefficient
$\tau_{sw}$	Stress at wall (N/$m^{2})$
$Re_{x}\;\;\;\;\;\;\;\;\;\;$	Reynolds number
$Nu_{x}$	Nusselt number
